# Cognitive behavioral group therapy for panic disorder in a general clinical setting: a prospective cohort study with 12 to 31-years follow-up

**DOI:** 10.1186/s12888-020-02679-w

**Published:** 2020-05-24

**Authors:** Truls Bilet, Torbjørn Olsen, John Roger Andersen, Egil W. Martinsen

**Affiliations:** 1 Division of Psychiatry, District General Hospital of Førde, Førde, Norway; 2 Centre of Health Research, Førde Hospital Trust, Førde, Norway; 3 Faculty of Health and Social Sciences, Western Norway University of Applied Sciences, Førde, Norway; 4 Division of Mental Health and Addiction, Oslo University Hospital, Oslo, Norway; 5 Institute of Clinical Medicine, University of Oslo, Oslo, Norway

**Keywords:** Anxiety, Panic disorder, Agoraphobia, CBT, Group therapy, Long term follow-up, Quality of life, Treatment satisfaction

## Abstract

**Background:**

The long-term prognosis after cognitive behavioral therapy (CBT) in outpatient groups for panic disorder and agoraphobia is not well known. The purpose of this study was to assess long-term outcomes in terms of psychological health, health-related quality of life (HRQoL), quality of life (QoL) and treatment satisfaction after CBT for panic disorder and agoraphobia.

**Methods:**

The sample consisted of 68 patients (61% response rate), who were assessed at pretreatment; at the start and end of treatment; and after 3 months, after 1 year, and over the long term (M = 24 years; SD = 5.3; range: 12 to 31 years). The main outcome was the total score on the Phobic Avoidance Rating Scale (PARS-total). At long-term follow-up, HRQoL was measured with the RAND-12 questionnaire, and QoL was measured with two questions from the “Study on European Union Statistics on Income and Living Conditions”. Patient experiences and treatment satisfaction were assessed by the Generic Short Patient Experiences Questionnaire. A marginal longitudinal model was applied to study the main outcome.

**Results:**

The effect size of the long-term change (mean change/ pooled SD) in the PARS-total score was (− 1.6, *p* < 0.001) and was stable over time. A PARS-total score reduction of 50% was found in 98% of patients at the long-term follow-up. The patients’ HRQoL and QoL were similar to the expected scores for the general Norwegian population. Of the patients, 95% reported high to very high satisfaction with the CBT, and 93% reported large treatment benefits.

**Conclusions:**

To the best of our knowledge, this study has the longest follow-up after group CBT for panic disorder and agoraphobia, showing a good prognosis in ≥93% of the participating patients.

## Background

Panic disorder and agoraphobia are disabling disorders often associated with impairments in a wide range of life areas [1]. The first documented treatment for agoraphobia was exposure therapy, and later, the cognitive elements of treatment were developed. The combined form of treatment including both exposure and cognitive therapy was labeled cognitive behavioral therapy (CBT), which is increasingly viewed as the treatment of choice [2]. Antidepressant medication use is well documented [3], and there is also empirical evidence for less traditional forms of treatment, such as physical exercise [4].

Several randomized controlled trials have shown good effects of CBT in the treatment of panic disorder with or without agoraphobia [2, 5]. In the majority of these studies, patients were selected with strict exclusion criteria, and therapists received specialized training. Fewer studies have been conducted in the general clinical setting, where patients have been treated by therapists without specialized therapeutic competence. CBT is most often delivered individually, and most scientific studies have addressed individual CBT. Some have found that good results can also be achieved following group CBT [6], and this format may be more cost effective and feasible in the clinical setting. Nevertheless, the number of studies, especially long-term studies (> 2 years), on CBT in groups is scarce [5, 7–14]. Long term outcome following CBT varies across studies. The first studies showed modest results, indicating that these disorders tend to follow a chronic course [9–12]. In two long-term studies with patients following inpatient treatment with emphasis of group therapy, 38 patients (71%) were assessed 20 years after a combination of exposure and psychodynamic therapy [13], while 31 (67%) were investigated after cognitive and guided mastery therapy [14]. The long-term outcomes were very good in both samples.

We found no long-term follow-up study of patients treated with group CBT in the general clinical setting. Moreover, a Cochrane review recommended that studies in this field include broader measures of quality of life (QoL) in addition to standard disease-specific outcomes [8]. Thus, we wanted to address these research needs by extending a prior study (*N* = 83) published in 1998 [15]. In that study, group CBT was offered to patients with panic disorder belonging to the catchment area of the outpatient clinic of the Central Hospital of Førde in Western Norway, with a rural population of 35,000 people. In general, the patients improved during treatment and maintained those gains at one-year follow-up.

The principal aim of the current study was to investigate the long-term trajectories of outcomes in panic disorder following outpatient CBT in groups in a general clinical setting by extending the 1998 sample and performing a long-term follow-up. The secondary aims were to investigate these patients’ long-term health-related quality of life (HRQoL) and QoL compared to those of the average general Norwegian population and to address patient experiences and treatment satisfaction.

## Methods

In this prospective naturalistic study, assessments were carried out by personal interviews at pretreatment, at the first and last treatment sessions, and at the 3-month, 1-year, and 12 to 31 years follow-up (mean follow-up time was 24-years; SD 5.3). The wide range of long-term follow-up time points is due to the merging of the sample from the 1998 study and new patients. The study was approved by the Regional Committee for Medical Research Ethics in Western Norway (REK) and is registered in the REK archive (reference number: 2016/2693) [16]. Patients were recruited from a regional outpatient clinic serving a catchment area of approximately 35,000 people referred from doctors in primary care or somatic departments. The treatment team received all referrals with a probable diagnosis of panic disorder. Those who met the DSM-III-R criteria for panic disorder with or without agoraphobia and did not meet the criteria for psychotic or posttraumatic stress disorders were offered CBT in a group format.

### Sample

Of the 183 eligible patients who received treatment in the period 1989–2008, we were not able to trace the addresses of 63 patients (34%), while 8 (5%) were deceased. Thus, we invited 112 patients to participate. Of these, 2 (1%) did not want to participate, 9 (5%) wanted to participate but were unable for different reasons, and 32 (17%) did not respond. The final sample consisted of 68 patients, yielding a response rate of 61%, and they met with the therapists at the hospital. The average follow-up time since treatment was 23.8 years (SD, 5.4), ranging from 12 to 31 years. The participant characteristics at the start of treatment are presented in Table 1.

**Table 1:** Pretreatment patient characteristics

Characteristics	Long-term participants (*n* = 68)	Non-long-term participants (*n* = 114)
Age, years, mean (SD)	34.8 (10.3)	37.6 (10.0)
Females, %	73.5	69.1
Marital status,%		
Married or cohabiting	69.1	76.3
Divorced/widowed	11.8	6.1
Single	19.1	17.6
Occupation, %		
Employed	68.7	64.0
Receiving social security benefits	13.5	18.5
Other, not employed	17.7	17.5
Age at onset of anxiety disorder, mean (SD)	28.0 (9.0)	30.6 (10.4)
Years of duration of anxiety disorder, mean (SD)	6.8 (7.4)	6.8 (8.0)
Previous psychiatric treatment, %	48.5	51.8
DSM-III-R Axis diagnosis, Panic disorder, %		
With agoraphobia	77.9	78.1
Without Agoraphobia	22.1	21.9

*SD* standard deviation

### Treatment program

Treatment was delivered in groups of 6 to 10 participants. The groups met each week for 11 four-hour sessions. The families or significant others were invited to one of the sessions. All group sessions were conducted by the same social worker (TO) assisted by various therapists who were either registered psychiatric nurses or registrars (TB and others). The patients in each group had two lectures on anxiety disorders and their treatment by EWM, who also supervised the therapists weekly. At the pretreatment stage, the patients were given a brief outline of the treatment explaining the rationale for exposure and cognitive restructuring. All patients who met the criteria for major depression during the pretreatment visit were offered antidepressants. Benzodiazepines were tapered and stopped within the first weeks of treatment. Patients were encouraged not to use alcohol while in therapy.

Initial sessions were devoted to psychoeducation with special emphasis on the connections between perceived threat, somatic symptoms of arousal, automatic thoughts, and anxious feelings. The physiological symptoms of sympathetic activation were examined in detail, as were feelings of disaster and catastrophic interpretations of somatic symptoms. The patients were assisted in identifying their own vicious cycles of symptoms, thoughts, and feelings and learned to rate their anxiety on a 0–10 scale. The patients were repeatedly encouraged to use diaries for recording anxiety ratings, noting daily homework assignments, discussing dysfunctional thoughts, and personally monitoring the gains made in therapy [17].

All participants underwent voluntary hyperventilation early in treatment. Behavioural experiments were used to some extent.

The positive benefits of regular physical activity were emphasized, and the ways in which physical activity influences health in general and anxiety specifically were explained. The patients were encouraged to implement a life-long habit of walking at least 30 min daily or undertaking other physical activities of their choice.

#### Group sessions consisted of four modules

1. Review of homework, including anxiety ratings during exposure, and discussion of somatic symptoms, dysfunctional thoughts, and coping strategies.
2. Planning, performing, and reviewing the present day’s individual in vivo exposure at downtown locations, such as attending public offices, riding the omnibus, shopping, walking the streets, or going to a café.
3. Review of the progress made during the exposure and recapitulation of cognitive theory.
4. Assignment of daily homework for the week to come

### Procedure

For the long-term follow-up investigation patients met in groups and were offered sandwiches, tea or coffee. Travel expenses were covered. The Phobic Avoidance Rating Scale (PARS) was scored in individual interviews. The other instruments were self-rating and filled in by the patients while sitting in the group.

### Measures

The main outcome is based on the Phobic Avoidance Rating Scale (PARS), an observer-rated scale that measures the degree of avoidance. Each item is scored on a 5-point scale where 0 represents no avoidance and 4 indicates total avoidance of the situation in question [18]. We defined a 50% reduction in the PARS-total score as the primary indicator of a substantial response to treatment. Reduced avoidance of this magnitude is readily recognized as clinically significant by both the patient and therapist.

Level of depression was assessed by the Beck Depression Inventory (BDI) [19], the degree of catastrophic interpretations of somatic symptoms by the Body Sensations Questionnaire (BSQ) [20], the fear of fear by the Agoraphobic Cognitions Questionnaire (ACQ) [20] and Agoraphobic Cognitions Scale (ACS) [21], and the degree of phobic avoidance by the Mobility Inventory for Agoraphobia (MIA) [22], with subscales for avoidance both alone and accompanied by others.

HRQoL, a multidimensional construct encompassing physical, psychological and social dimensions of health [23], was measured with the RAND-12 questionnaire, which covers 12 dimensions of functioning and well-being [24]. The RAND-12 has two summary scores: the physical component summary (PCS) and mental component summary (MCS). To calculate the summary scores, we applied a formula allowing the PCS and MCS to be freely correlated [24]. Higher PCS and MCS represent better scores. RAND-12 scores from the general population were obtained from the Norwegian Survey on Living Conditions in 2002 (*N* = 5396) [25].

QoL, a global construct encompassing an overall assessment of well-being or life satisfaction [23], was assessed by two questions: (1) Life satisfaction: “Overall, how satisfied are you with your life nowadays (where zero is not at all satisfied and 10 is completely satisfied)?” (2) Life meaning: “Overall, to what extent do you feel that the things you do in your life are worthwhile (where zero is not at all worthwhile and 10 is completely worthwhile)?” QoL scores from the general population were obtained from the Norwegian part of the European Union Statistics on Income and Living Conditions (EU-SILC) Survey in 2017 (*N* = 6168) [25].

Patient experiences and treatment satisfaction were assessed by the Generic Short Patient Experiences Questionnaire (GS-PEQ), which consists of ten generic core questions that cover the essential dimensions of users’ experiences with a range of specialist health care services [26]. The ten GS-PEQ item scores are intended to be used as single indicators for each of the ten specific content areas.

HRQoL, QoL and patient experiences were assessed only at long-term follow-up.

### Statistics

Data are presented as frequencies or percentages and as means and standard deviations or 95% confidence intervals. Exact two-sided *p*-values are reported. Longitudinal marginal models with an unstructured covariance structure were used in analyses of change, with time as a fixed categorical effect. This method handles missing y-data by estimating outcomes based on all available data. The length of time since starting treatment was not correlated with long-term PARS-total scores (r = 0.06, *p* = 0.642). Thus, we coded all long-term data (ranging from 12 to 31 years) into one category. The trajectories of the PARS-total score during the first year of follow-up for long-term participants versus non-long-term participants were displayed, and the interaction effect of time*group was tested. Age- and gender-adjusted comparisons of generic health status and QoL between the patient group and the general population were conducted with a one-sample t-test. Effect sizes of changes over time were calculated by using change scores from the longitudinal marginal models divided by the crude pooled standard deviations at the start of treatment and at the last time point. Effect sizes for score differences in the patient group compared to the general population were calculated by using difference scores divided by the standard deviations in the patient group. Effect sizes were interpreted according to the following criteria: trivial (< 0.2), small (0.2 to < 0.5), moderate (0.5 to < 0.8 SD), and large (≥0.8) [27]. The statistical analyses were conducted using SPSS, version 25.0 (SPSS Inc., Chicago, USA).

## Results

**Fig. 1:**
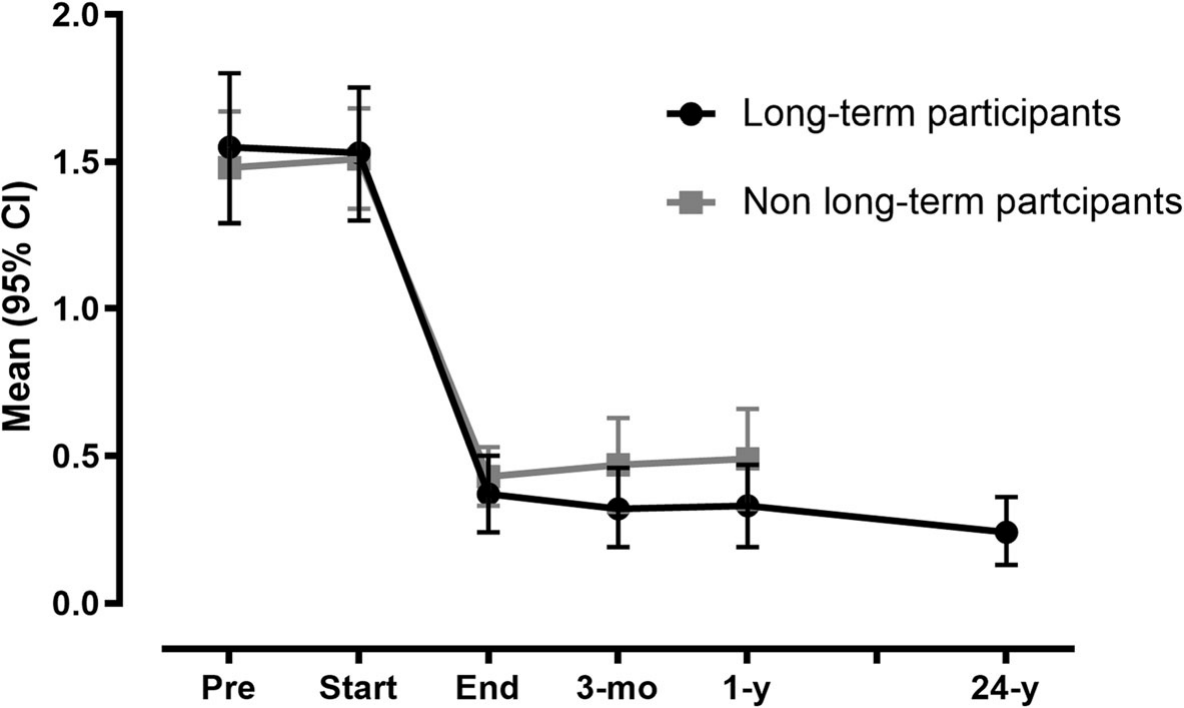
Mean total Phobic Avoidance Rating Scale trajectories over time in patients attending the 24-year follow-up (long-term participants) versus the other patients (non-long-term participants). Pre: pretreatment, Start: start of treatment, End: end of treatment, 3-mo: 3-month follow-up, 1 y: 1-year follow-up, 24 y: mean follow up time = 24-years, range 12–31 years. *P*-value for time * group interaction = 0.647

The PARS score was stable from pretreatment to the start of treatment. The PARS-total score improved during treatment (*p* < 0.001) (Table 2). The effect size was large at the end of treatment and remained stable thereafter. A reduction of 50% in the PARS-total score was found in 82% of the patients by the end of treatment and in 98% of patients by the 24-year follow-up. All secondary outcomes also improved over time (Table 2; *p* < 0.001 for all). The standard deviations for all scores decreased over time, indicating that the response to treatment was general. There were no significant differences between the patients who were assessed at the 24-year follow-up and the other patients in terms of their PARS-total trajectories over time (*p* = 0.647) (Fig. 1).

**Table 2:** Means scores based on marginal models with crude standard deviations before and after cognitive behavioral group therapy

Rating scale	Pretreatment (*n* = 139)	Start of treatment (*n* = 173)	End of treatment (*n* = 170)	3-month follow-up (*n* = 142)	1-year follow-up (*n* = 118)	Long-term follow-up (*n* = 68)	Effect size^a^
**PARS-tot.**	**1.5 (0.9)**	**1.5 (0.9)**	**0.4 (0.5)**	**0.4 (0.7)**	**0.4 (0.7)**	**0.3 (0.5)**	−1.6
PARS-sep.	1.6 (1.0)	1.5 (1.0)	0.4 (0.5)	0.3 (0.6)	0.4 (0.7)	0.3 (0.5)	−1.6
PARS-soc.	1.5 (1.1)	1.5 (1.1)	0.4 (0.7)	0.5 (0.8)	0.5 (0.8)	0.3 (0.5)	−1.4
MI-AAC	50.5 (20.1)	50.6 (19.3)	34.4 (11.0)	33.7 (10.8)	33.3 (13.1)	31.4 (7.2)	−1.3
MI-AAL	69.2 (25.0)	67.8 (27.2)	42.3 (15.8)	41.2 (15.6)	40.5 (19.4)	37.5 (14.5)	−1.6
ACQ	37.0 (10.1)	37.8 (11.1)	25.9 (9.0)	24.1 (9.0)	22.9 (7.6)	21.8 (7.7)	−1.7
BSQ	47.3 (13.6)	46.6 (12.4)	29.9 (10.5)	28.9 (11.1)	28.4 (12.2)	28.2 (11.5)	−1.5
BDI	16.7 (8.2)	15.5 (8.6)	7.4 (6.8)	7.3 (8.1)	6.5 (8.1)	5.9 (5.9)	−1.5

Long-term follow-up (mean = 24 years; range = 12–31 years)

*Abbreviations: ACQ* Anxiety Cognitions Questionnaire, *ACS* Agoraphobic Cognitions Scale, *BDI* Beck Depression Inventory, *BSQ* Body Sensation Questionnaire, *MI-ACC* Mobility Inventory for Agoraphobia, Avoidance Accompanied, *MI-ALL* Mobility Inventory for Agoraphobia, Avoidance Alone, *PARS* Phobic Avoidance Rating Scale, *PARS-sep* PARS Separation Subscale, *PARS-soc* PARS Social Subscale

*P*-values were < 0.001 for the effect of time on all scales

^a^ Effect size: (Long-term follow-up – Pretreatment / pooled standard deviation)

The patients’ HRQoL and QoL 24 years after CBT were similar to the expected scores in the general population after adjustment for age and gender (Table 3). Details on patient experiences and treatment satisfaction at the 24-year follow-up are presented in Table 4. The patients had high ratings on all items, with 95% reporting high to very high satisfaction with the CBT and 93% reporting large benefits.

**Table 3:** General health status and quality of life in the patient group at 12 to 31-years follow-up compared to the general Norwegian population

Outcomes	Patient mean (SD)	General population mean	*P*-value	Effect size
Physical health status	49.5 (9.4)	49.4	0.923	0.1
Mental health status	48.8 (11.4)	51.1	0.104	−0.3
Life satisfaction	8.0 (2.1)	8.1	0.699	−0.1
Life meaning	8.3 (1.7)	8.1	0.413	0.2

Differences in outcomes are adjusted for age and gender. Effect size = mean difference in scores between patients and the general population divided by the standard deviation of the patient scores

**Table 4:** The Generic Short Patient Experiences Questionnaire scores at 12 to 31-years follow-up

Items	Not at all	To a small extent	To a moderate extent	To a large extent	To a very large extent	Not applicable
Did the *clinicians* talk to you in a way that was easy to understand?	0	0	1	26	31	0
Do you have confidence in the *clinicians’* professional competence?	0	0	1	14	43	0
Did you get sufficient information about your diagnosis/your afflictions?	0	0	5	26	27	0
Did you perceive the treatment you received as suited to your situation?	0	0	4	21	33	0
Were you involved in any decisions regarding your treatment?	0	2	6	21	28	1
Did you perceive the institution’s work as well organized?	0	0	1	24	31	2
Do you believe that you were in any way given the wrong treatment (according to your own judgment)?	49	4	1	1	0	3
Overall, were the help and treatment you received at the institution satisfactory?	1	0	1	17	36	1
	No	Yes, but not so long	Yes, quite long	Yes, much too long	–	Not applicable
Did you have to wait before you were admitted for services at the institution?	27	25	3	1		0
	No benefit	Small benefit	Some benefit	Great benefit	Huge benefit	Not applicable
Overall, what benefit have you had from the care at the institution?	0	1	3	20	33	0

The results are presented as the distribution in scores according to the crude numbers of patients on the different answer categories (number of patients ranging from 56 to 58)

## Discussion

To the best of our knowledge, this study has the longest follow-up after group CBT for panic disorder and agoraphobia. The effect size for clinical treatment was large at the end of treatment and remained stable thereafter. A 50% reduction in the PARS-total score was found in 98% of patients at the long-term follow-up, and 93% of patients were satisfied with the outcomes. The patients’ HRQoL and QoL were similar to the expected scores for the general Norwegian population.

Our results correspond well with previous shorter-term studies on the treatment of panic disorder and agoraphobia [2, 4]. The few studies reporting long-term outcomes have diverging results, and the explanations for this is not clear. Our results correspond best to the previous studies with longest follow-up [13, 14]. An interesting common feature of these studies is the focus on the group format. Our clinical experience indicate that the group fellowship was important for many patients. Systematic exposure treatment and cognitive therapy seem to yield substantial and lasting therapeutic gains, and these results can also be achieved in the general clinical setting. Thus, the good results following individual CBT seem to be achieved by group CBT as well. This format is suitable for training new therapists, who can learn the method by working as co-therapists with more experienced professionals.

The main strength of this study is its length of follow-up. Moreover, the study was conducted in the general clinical setting, had few exclusion criteria and included participants with multiple comorbidities. Thus, the results are generalizable to the general clinical setting. Furthermore, we had a broad set of validated measures encapsulating mental health, HRQoL, QoL, and patient experiences and treatment satisfaction. The study’s main limitations are its observational design, and we do not know the long-term prognosis of the 39% of patients who did not participate in the long-term follow-up. Loss of patients is also observed in other studies with a very long follow-up period [13, 14]. Patients may have chosen not to participate in the long-term follow-up because they were doing well and perceived their attendance as unnecessary, because they were doing poorly and did not want further contact, or because they had other reasons unrelated to the study outcomes. However, it is reassuring that the PARS-total trajectories during the first year after treatment were similar in long-term -and other participants. Unfortunately, we have no measure of remission, only of responders. It would also had been nice to have a therapist rating which covered panic/agoraphobia in a broader sense than only avoidance, but these other aspects are covered by self- report instruments.

## Conclusions

Our study suggests that CBT in groups is feasible and that the therapeutic gains last for a very long period of time. This finding is good news for a common mental health problem. However, further studies are needed to establish a robust body of evidence on this matter.

## Data Availability

The dataset generated during this study is not publicly available, as the patient consent and approval from the Regional Committee for Medical and Health Research Ethics prevent us from sharing individual patient-level data in public repositories. However, the data are available upon reasonable request from the corresponding author.

## Abbreviations

CBT: Cognitive Behavioral Therapy
HRQoL: Health-Related Quality of Life
QoL: Quality of Life
RARS: Phobic Avoidance Rating Scale
REK: Regional Committee for Medical Research Ethics in Western Norway
BDI: Beck Depression Inventory
BSQ: Body Sensations Questionnaire
ASQ: Agoraphobic Cognitions Questionnaire
ACS: Agoraphobic Cognitions Scale
MIA: Mobility Inventory for Agoraphobia
PCS: Physical Component Summary
MCS: Mental Component Summary
EU-SILC: European Union Statistics on Income and Living Conditions
GS-PEQ: Generic Short Patient Experiences Questionnaire
